# Supplementary material for: Coinfection Ecology and Pathogen Emergence in a Borrelia-Endemic Landscape: Five Years of Borrelia burgdorferi, Anaplasma phagocytophilum, and Babesia microti Surveillance in Maryland
Source: medRxiv. 2025 Dec 4:2025.12.03.25341558. Preprint. [Version 1] doi: 10.64898/2025.12.03.25341558 (PMC12706617; doi:10.64898/2025.12.03.25341558)
Supplement: 1 [file NIHPP2025.12.03.25341558V1-supplement-1.pdf]

medRxiv preprint doi: https://doi.org/10.64898/2025.12.03.25341558; this version posted December 4, 2025. The copyright holder for this preprint (which was not certified by peer review) is the author/funder, who has granted medRxiv a license to display the preprint in perpetuity.

It is made available under a CC-BY 4.0 International license .

SUPPLEMENTARY MATERIAL

Pathogen	Tick Source	Site	n Tested	n Positive	OR per Year	95% CI	p-value
Anaplasma	All ticks	Statewide	557	21	1.12	0.79–1.59	0.5369
Anaplasma	All ticks	EHH	165	8	1.39	0.71–2.70	0.3362

medRxiv preprint doi: https://doi.org/10.64898/2025.12.03.25341558; this version posted December 4, 2025. The copyright holder for this preprint (which was not certified by peer review) is the author/funder, who has granted medRxiv a license to display the preprint in perpetuity.

It is made available under a CC-BY 4.0 International license .

Pathogen	Tick Source	Site	n Tested	n Positive	OR per Year	95% CI	p-value
Anaplasma	All ticks	GFH	225	7	0.81	0.49–1.35	0.4191
Anaplasma	All ticks	HHB	167	6	1.84	0.67–5.03	0.2360
Babesia	All ticks	Statewide	557	55	1.33	1.04–1.69	0.0221
Babesia	All ticks	EHH	165	14	1.52	0.98–2.36	0.0637
Babesia	All ticks	GFH	225	12	1.07	0.71–1.61	0.7395
Babesia	All ticks	HHB	167	29	1.35	0.91–2.01	0.1326
Anaplasma	Drag only	Statewide	333	9	1.42	0.86–2.35	0.1710
Babesia	Drag only	Statewide	333	11	1.58	1.01–2.49	0.0462
Anaplasma	Peromyscus-fed	Statewide	224	12	1.02	0.67–1.57	0.9154
Babesia	Peromyscus-fed	Statewide	224	44	1.21	0.95–1.54	0.1142
Anaplasma	Peromyscus-fed	EHH	54	6	1.49	0.83–2.69	0.1760
Anaplasma	Peromyscus-fed	GFH	112	4	0.69	0.35–1.34	0.2655
Anaplasma	Peromyscus-fed	HHB	58	2	1.68	0.50–5.63	0.4017
Babesia	Peromyscus-fed	EHH	54	8	1.73	1.05–2.86	0.0316
Babesia	Peromyscus-fed	GFH	112	4	1.06	0.66–1.72	0.8086
Babesia	Peromyscus-fed	HHB	58	15	1.30	0.88–1.93	0.1908

Supplementary Table 1. Logistic Regression of Infection Prevalence by Year: Logistic regression results testing for monotonic changes in infection prevalence over time (2020–2024), stratified by pathogen, tick type, and site. OR = Odds Ratio; CI = Confidence Interval.

County	Anaplasmosis 2019	2020	2021	2022	2023	Babesiosis 2019	2020	2021	2022	2023
Allegany	0	0	0	0	0	0	0	0	0	0
Anne Arundel	0	1	3	1	1	0	0	0	0	3
Baltimore City	5	0	0	1	4	0	1	1	0	2
Baltimore County	2	0	0	0	0	2	2	2	2	0
Calvert	1	0	0	2	0	0	0	0	0	0
Caroline	0	0	0	0	0	0	0	0	0	0
Carroll	1	0	2	1	2	1	0	2	1	2
Cecil	1	1	0	0	1	0	0	1	0	0
Charles	0	0	0	0	0	0	0	0	0	0
Dorchester	0	0	0	0	2	0	0	0	0	0
Frederick	1	0	1	0	1	0	0	2	0	2

medRxiv preprint doi: https://doi.org/10.64898/2025.12.03.25341558; this version posted December 4, 2025. The copyright holder for this preprint (which was not certified by peer review) is the author/funder, who has granted medRxiv a license to display the preprint in perpetuity.

It is made available under a CC-BY 4.0 International license .

County	Anaplasmosis 2019	2020	2021	2022	2023	Babesiosis 2019	2020	2021	2022	2023
Garrett	0	0	0	1	2	0	0	0	0	0
Harford	0	0	0	1	4	1	0	0	0	2
Howard	1	0	2	2	0	1	1	1	0	3
Kent	0	0	0	0	0	0	0	0	0	0
Montgomery	2	4	6	6	3	1	3	3	2	8
Prince George's	0	0	0	1	2	0	1	0	0	5
Queen Anne's	0	0	0	0	0	0	0	0	0	0
St. Mary's	0	0	0	0	0	0	0	0	0	0
Somerset	0	0	0	1	0	0	0	0	0	0
Talbot	0	0	0	0	1	0	0	0	0	1
Washington	1	0	0	0	1	0	0	0	0	0
Wicomico	1	0	1	0	2	0	0	0	1	0
Worcester	0	0	2	0	0	0	1	1	1	1

Supplementary Table 2. Confirmed Human Cases of Anaplasmosis and Babesiosis in Maryland by County, 2019–2023: Case counts were obtained from Maryland Department of Health public surveillance data
